# Identification and Validation of Reference Genes for Expression Analysis Using RT-qPCR in *Leptocybe invasa* Fisher and La Salle (Hymenoptera: Eulophidae)

**DOI:** 10.3390/insects14050456

**Published:** 2023-05-12

**Authors:** Ya Liu, Jing Zhou, Zhisong Qiu, Ping Hu, Xiao Chen, Zhende Yang

**Affiliations:** 1 College of Forestry, Guangxi University, Nanning 530004, China; 2 Guangxi Key Laboratory of Forest Ecology and Conservation, State Key Laboratory for Conservation and Utilization of Subtropical Agro-Bioresources, College of Forestry, Guangxi University, Nanning 530004, China

**Keywords:** *Leptocybe invasa*, target genes, reference genes, gene stability, RT-qPCR

## Abstract

**Simple Summary:**

In gene expression investigations, the first crucial step is choosing appropriate housekeeping genes. However, the choice of reference genes is not absolute but relative and varies with different experimental conditions. It is vital to note that using unvalidated or unscreened internal reference genes can lead to erroneous inferences. This study was conducted on *Leptocybe invasa* to calculate the stability of eight housekeeping genes across various test conditions, such as sexes, somites, temperatures, diets, and pesticides. The relative expression of *HSP90* at different temperature settings was evaluated to validate the results. This study aims to assist future gene expression research on this invasive species and lay the groundwork for further investigations into the gene function of this pest.

**Abstract:**

*Leptocybe invasa* (Hymenoptera: Eulophidae) is a globally intrusive pest. Despite extensive research into the physiological responses of this pest, our understanding of the molecular mechanisms still needs to be improved. We want to accurately investigate the expression of *L. invasa*’s target genes, so it is imperative to select fitting reference genes. In this study, eight housekeeping genes’ stability (*RPS30*, *ACTR*, *18S rRNA*, *ACT*, *RPL18*, *GAPDH*, *28S rRNA*, and *TUB*) was tested under five different experimental conditions, including male or female adults, somites (head, thorax, and abdomen), temperatures (0 °C, 25 °C, and 40 °C), diets (starvation, clear water, 10% honey water, *Eucalyptus* sap), and pesticides (acetone was used as a control, imidacloprid, monosultap). Gene stability was calculated using RefFinder, which integrates four algorithms (the ∆Ct method, geNorm, NormFinder, and BestKeeper). The findings implied that *ACT* and *ACTR* were the most accurate when comparing sexes. For analyzing different somites, *28S rRNA* and *RPL18* were ideal; the *28S rRNA* and *RRS30* were perfect for analyzing at different temperatures. The combination of *ACT* and *GAPDH* helped to analyze gene expression in different diets, and *GAPDH* and *28S rRNA* were suitable for various pesticide conditions. Overall, this research offers a complete list of reference genes from *L. invasa* for precise analysis of target gene expression, which can improve the trustworthiness of RT-qPCR and lay the foundation for further investigations into the gene function of this pest.

## 1. Introduction

The *Eucalyptus* tree is Australia’s national tree and one of the southern hemisphere’s most crucial fast-growing tree species for industries [1]. *Leptocybe invasa* Fisher and La Salle (Hymenoptera: Eulophidae) is a pest that infests *Eucalyptus* woodlands worldwide. Since its initial detection in the Middle East and the Mediterranean in 2000, *L. invasa* has caused significant damage to nurseries and young *Eucalyptus* forests. Moreover, it has quickly spread to many *Eucalyptus*-growing nations, such as Australia, China, India, and Brazil [2,3]. *L. invasa* has colonized 45 countries and regions across five continents, including Oceania, Asia, and Europe [4]. The presence of large numbers of galls in *Eucalyptus* plantations and young forest nurseries not only raises concerns about the quality of damaged *Eucalyptus* trees but also increases the likelihood of significant economic damage to the region’s *Eucalyptus* forests [5]. Although many studies were performed on the pattern of *L. invasa* proliferation, genetic diversity, and in vivo bacterial diversity [4,6,7], little is known about the molecular processes in *L. invasa* gene expression. Recently, by analyzing the transcriptome of *L. invasa*, we identified a significant number of heat shock protein genes and some resistance genes. We want to learn more about the expression patterns of these genes and how they function to control the pest in the future better. However, *L. invasa* internal reference genes have not yet been screened or used.

The internal reference genes are the foundation for investigating the insect target gene’s expression. Real-time fluorescence quantitative PCR (RT-qPCR) has emerged as a highly delicate and sophisticated technique widely adopted for analyzing low-abundance mRNA expression levels. This technique has been acknowledged for its exceptional stability, precision, efficiency, and speed in mRNA quantification, making it the preferred method for examining gene expression levels in various biological contexts [7,8]. Systematic errors during RT-qPCR analyses can occur during RNA extraction, polymerase amplification, and cDNA synthesis [8,9]. Using housekeeping genes as controls is an integral step to ensure accurate measurement of gene expression levels and enable valid comparisons between different samples. Standardizing mRNA levels across many samples is critical for obtaining reliable gene expression data in various experimental settings [10]. To ensure gene expression studies’ steady and accuracy, these benchmark genes must maintain a high level of stability in their expression throughout multiple stages of organism development, under different treatments and environmental conditions, and across diverse cell or tissue types [11,12]. In studies on internal insect reference gene screening, 18S ribosomal RNA (*18S rRNA*), ribosomal protein S18 *(PRS18*), beta-tubulin (*TUB*), and glyceraldehyde 3-phosphate dehydrogenase (*GAPDH*) are commonly mentioned [13,14]. These genes are all engaged in the typical physiological and metabolic activities of cells and are frequently utilized as internal reference genes [7,15]. A perfect reference gene should continue to express at a similar level even when subjected to various experimental conditions [16]. Yet, many researchers have used RT-qPCR to analyze the internal reference genes, and the results demonstrate that no gene can exhibit steady expression in various experimental conditions. Benchmark genes for RT-qPCR analysis depend on the specific experimental context [15,17]. In the case of *Anastatus japonicus* development, *RPS6* and *RPL13* were determined to be perfect. Meanwhile, when analyzing adults of different sexes, *ACTIN* and *EF1α* were perfect. When analyzing diverse tissues, *RPL13* and *EF1α* performed better than other genes. Finally, *TATA* and *ACTIN* were recognized as excellent for evaluating distinct diapause conditions [17]. *RPS18* and EF1α were the two trustiest genes in *Neoceratitis asiatica*, whereas *RPS15* and *EF1β* were the most untrustworthy [18]. Instead of employing generic reference genes, choose appropriate housekeeping genes under the circumstances for specific insect species. Therefore, it is indispensable to ascertain the optimum housekeeping gene for *L*. *invasa* conditions.

The main aim of the current investigation was to ascertain the finest housekeeping genes for RT-qPCR analysis in *L. invasa* under various conditions, such as sex, somite, temperature, diet, and pesticide, by using five statistical techniques (comparative ∆Ct [9], geNorm [19], NormFinder [20], BestKeeper [21], and RefFinder [22]) for standardization. Furthermore, the average relative expressions of heat shock protein 90 (*HSP90*) were analyzed to authenticate the effectiveness of the housekeeping gene. The findings reported in this research endeavor are poised to galvanize further inquiry into the gene expression of *L. invasa*, an invasive species. Such investigations will play a pivotal role in augmenting our comprehension of the underlying molecular pathways that drive the stress response mechanisms in this species.

## 2. Materials and Methods

### 2.1. Insect Rearing and Plant Preparing

*L. invasa* was taken in 2021 from Nanning in the Guangxi Zhuang Autonomous Region (22.48 °N, 108.22 °E) and raised on *Eucalyptus grandis × Eucalyptus tereticornis* (DH201-2) until galls developed. *Eucalyptus grandis × Eucalyptus tereticornis* (DH201-2) was earlier grown in a greenhouse of the Guangxi Forestry Research Institute, Nanning, Guangxi, China, without any pest or pesticide treatment and used for feeding insects when grown to seedling stage. All insects were housed in MGZ light incubators (Shanghai Binglin Electronic Technology Co., Ltd., Shanghai, China) at 26 ± 1 °C, light intensity 1800 lx, photoperiod 16L:8D. Except where otherwise indicated, the temperature and lighting conditions used in the following experiments were identical to those used during rearing. *L. invasa* was raised on *Eucalyptus grandis × Eucalyptus tereticornis* (DH201-2), a species relatively sensitive to *L. invasa*.

### 2.2. Experimental Treatments

#### 2.2.1. Different Sex

Male and female adult *L. invasa* newly emerged from DH201-2 of *Eucalyptus grandis × Eucalyptus tereticornis* were randomly collected, one replicate for every 60 male and female adults, for a total of three biological replicates. All insects were conserved in 1.5 mL RNAase-free centrifuge tubes with RNA preservation solution for *L. invasa*. All samples were left all night at 4 °C and then put at −20 °C pending the extraction of RNA. This approach was employed to collect and preserve samples without further specific descriptions in the following experimental treatments. Three biological replicates were set up during each of the subsequent experiments.

#### 2.2.2. Adult Somite

First, place a sterile Petri dish on ice, cover the Petri dish with a layer of sterile filter paper, and cut off the head, thorax, and abdomen of the adult worms with a special scalpel. The adult head, thorax, and abdomen were placed into three RNAase-free centrifuge tubes containing an RNA preservation solution. A total of 500 adult worms were dissected.

#### 2.2.3. Temperature Treatments

We collected newly emerged *L. invasa* adults from *Eucalyptus grandis × Eucalyptus tereticornis* DH201-2, and we kept all adults at 25 °C for 4 h. Afterwards to prevent the adults from dying of starvation, the adults were sited individually in 1.5 mL centrifuge tubes with 2 µL of 10% honey water in each tube, then 1 h at 0 °C, 25 °C, and 40 °C in MGZ light incubators, with 100 *L. invasa* per replicate.

#### 2.2.4. Diet Treatments

Adults were starved for 4 h after emergence and divided into four treatment groups: (i) no food as a control, (ii) water, (iii) a 10% honey solution, and (iv) diluted *Eucalyptus grandis × Eucalyptus tereticornis* DH201-2 sap. Samples were collected after 6 h. Sixty adults were used as a replicate.

#### 2.2.5. Pesticide Treatments

*L. invasa* adults were collected on the day of fledged, then the adults were sited in 1.5 mL centrifuge tubes, one head per tube, and each tube was filled with 2 µL of 10% honey water on the cap and fed for 3 h to avoid starvation. Then two commonly used pesticides for the control of *L. invasa* were selected, imidacloprid and monosultap. About 200 mg/mL film tubes were made by dissolving the drug in acetone. We introduced *L. invasa* into the drug film tubes for 1 h. One hundred *L. invasa* were used as one replicate. Acetone film tubes were controls.

### 2.3. RNA Extraction and cDNA Synthesis

TRIzol (Tiosbio, Beijing, China) and the instructions from the RNeasy Plus Mini Kit were used to quickly extract RNA from *L. invasa* (No. 74134; Qiagen, Hilden, Germany). Our analysis of the abstraction RNA was carried out using 1% agarose gel to verify its integrity. The concentration and pureness of RNA were determined by a NanoDrop 8000 spectrophotometer (Thermo Fisher Scientific, Waltham, MA, USA). A range of absorbance ratios was observed for the RNA samples at A_260/280_ and A_260/230_. Both are around 2.0, indicating that they are appropriate for future research. Based on instructions from TransScript One-Step gDNA Removal and cDNA Synthesis SuperMix (TranGen Biotech, Guangzhou, China), the first strand of cDNA was generated from each sample set. The resulting cDNA was then diluted by 20 µL for RT-qPCR. For the RT-qPCR, we maintained total RNA at −80 °C while keeping the complete cDNA at −20 °C.

### 2.4. Reference Gene Selection and Primer Design

In this study, based on the transcriptome data of *L. invasa*, many candidate reference genes were initially screened based on functional annotations. Then the candidate reference genes were further screened based on the FPKM value (FPKM > 50, medium expression is optimal and has similar expression levels in different samples), CV (CV < 0.15), and log2 fold value (absolute value less than 0.2) between samples. The corresponding gene sequences were found and then Blast compared on NCBI to homologous genes of other insects with 90% sequence similarity, which were used as candidate reference genes. We designed 18 primer pairs (product length 90–300) based on the CDS sequences of the corresponding genes and subsequently verified the stability through semi-quantitative RT-PCR and RT-qPCR. A total of eight primer pairs were selected for subsequent experiments. Eight internal reference genes were ribosomal protein S30 (*RPS30*), actin-related protein (*ACTR*), 18S ribosomal RNA (*18S rRNA*), actin (*ACT*), ribosomal protein L18 (*RPL18*), glyceraldehyde 3-phosphate dehydrogenase (*GAPDH*), 28S ribosomal RNA (*28S rRNA*), and B-tubulin (*TUB*). Using the web software Primer 3.0, primer pairs for amplification were created carefully by the RT-qPCR primer design guidelines with primer lengths of 20–22 bases, annealing temperature between 54 and 56 °C, and amplification product length greater than 90 bp and less than 300 bp [23,24]. DynaScience Biotechnology generated each primer in Table 1 (Beijing, China). Electrophoresis was performed on a 1% agarose gel to confirm the correctness of each primer. The sequences, lengths, and amplification efficiencies (E) of the eight benchmark genes’ primers are provided in Table 1.

### 2.5. RT-qPCR

RT-qPCR was performed using a LightCycler^®^ 480II Real-Time PCR System in 96-well plates (Roche Molecular Systems, Germany). Using Genious 2X SYBR Green Quick qPCR Mix (No ROX), the cDNA was amplified (ABclonal Technology, Woburn, MA, USA). The total cDNA template was subjected to a 5-fold gradient dilution to obtain cDNA templates at 5^0^, 5^−1^, 5^−2^, 5^−3^, and 5^−4^ ng.µL^−1^ concentrations for gradient concentration standard curve plotting. A 20 µL reaction system was used: Genious 2X SYBR Green Fast qPCR Mix 10 µL, forward and reverse primers 0.4 µL, cDNA template 1 µL, and ddH_2_O supplemented. We performed the RT-qPCR reaction in a 3-step standard reaction mode: 3 min pre-denaturation at 95 °C, 5 s denaturation at 95 °C, 30 s annealing, and extension at 60 °C, 40 cycles; 15 s at 95 °C, 60 s at 60 °C, and 15 s at 95 °C to form a melting curve. Each cDNA sample was subjected to three technical replicates, three biological replicates, and parallel inclusion of template-free controls. The relationship between Ct values and logarithmic cDNA template concentrations was analyzed using SPSS20 software, with the latter taken as the horizontal coordinate and the former as the vertical coordinate. To quantify the linearity of this relationship, we estimated the linear equation’s slope and regression coefficient (R^2^). According to the formula, the amplification efficiency (*E*) values were obtained [25,26].

$$E=\left(10^{-\frac{1}{slope}}-1\right)*100\%$$

### 2.6. Analyzing Reference Genes and Handling Data

The stability of eight benchmark genes was valued in diverse conditions using analysis and screening tools, including ∆Ct, geNorm, NormFinder, and BestKeeper. Furthermore, the online tool RefFinder (https://blooge.cn/RefFinder/?type=reference, accessed on 18 January 2023) was employed to comprehensively rank all housekeeping genes. While the original quantized cyclic values (Ct) can satisfy the criteria for the BestKeeper and comparative ∆Ct algorithms, for geNorm and NormFinder Analysis, the actual Ct values must be transformed to relative quantities. This part of the data was plotted using Origin 2021 (OriginLab, Northampton, MA, USA).

### 2.7. Verification of Reference Gene Stability

Heat shock proteins are a current research hotspot because they are widely distributed throughout most animals and are highly conserved. These heat shock proteins repair damaged proteins in response to heat or cold stimuli to sustain the organism’s regular life activities [26]. The accuracy of our experimental findings was further corroborated by the expression of the heat shock protein (*HSP90*) gene in *L. invasa* standardized by the two most optimal (*28S rRNA* and *RPS30*) and least reliable (*RPL18* and *TUB*) reference genes in different temperature, with 25 °C serving as the control. Using the 2^−∆∆Ct^, the relative expression levels of *HSP90* at various temperatures were calculated [27]. The expression levels of genes in diverse dealings were examined using one-way ANOVA, and the results were compared using Tukey’s highly significant difference test (Tukey’s HSD). This section uses GraphPad Prism 9 (GraphPad, San Diego, CA, USA) to process and plot the data.

## 3. Results

### 3.1. RNA Quality and Amplification Efficiency

The putative internal reference genes *28S rRNA*, *TUB*, *RPS30*, *ACTR*, *18S rRNA*, *ACT*, *RPL18*, and *GAPDH* were chosen based on the transcriptome analysis findings. Sequencing matching showed a greater than 90% sequence similarity with the same genes from other insects. Additionally, for the eight benchmark genes of *L. invasa*, the match’s expected value (E) was 0 (or nearly 0), indicating a perfect match for the genes, which also shows the highly conserved nature of these internal benchmark genes. The Ct values for the eight benchmark genes were significantly correlated with the cDNA values at various concentration gradients (*p* ≤ 0.001, 0.988 ≤ R^2^ ≤ 0.998, Table 1). A distinct single peak on the RT-qPCR solubility plots confirmed the specificity of the primers. These genes’ amplification effectiveness (E) values varied from 93% to 114%, with R^2^ > 0.990.

### 3.2. Levels of Expression of Reference Genes

**Figure 1:**
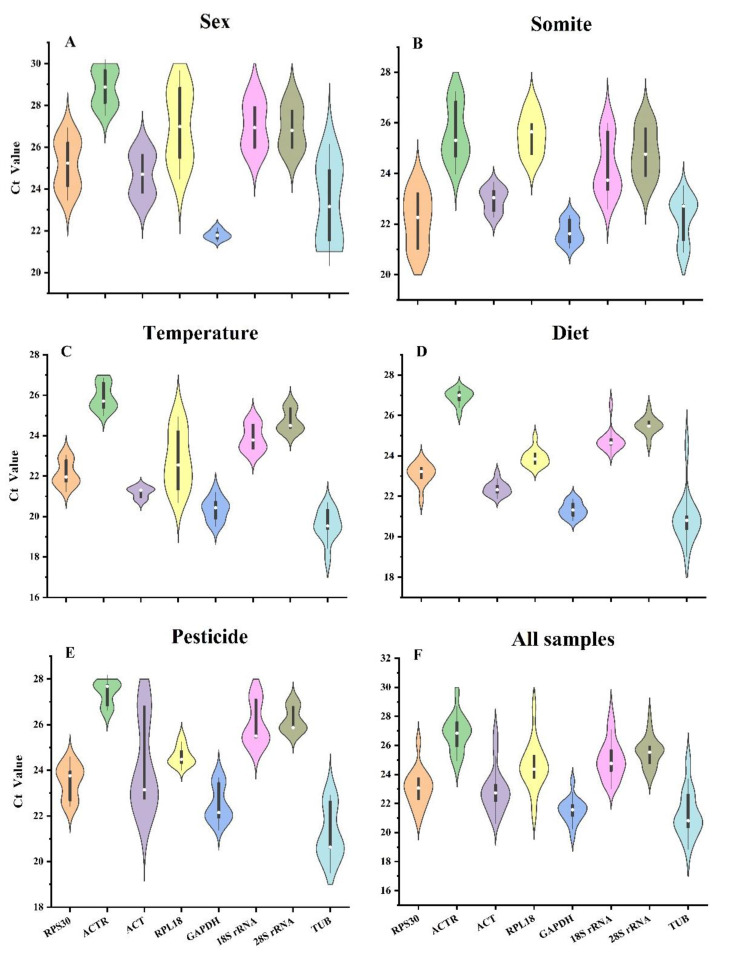
Reference gene expression levels under various experimental conditions: (**A**) sex, (**B**) somite, (**C**) temperature, (**D**) diet, (**E**) pesticide, and (**F**) all samples. The violin diagram’s white dot depicts the median Ct value, while the black bar indicates the interquartile range. The width of the violin is the richness of this set of data at this value of the vertical coordinate (frequency of each *y*-axis data). The different colors in the six violin diagrams represent different genes in the same order, from left to right, *RPS30*, *ACTR*, *ACT*, *RPL18*, *GAPDH*, *18S rRNA*, *28S rRNA*, and *TUB*.

The violin plot combines a bar chart (with the median as a white dot in the center) with a kernel density plot to provide a visual representation of the probability distribution of the data. The size of the area in the plot corresponds to the likelihood of the data being distributed around a certain value. Unlike a box line plot, the violin plot can show and more accurately represent the data distribution. The cycle of quantification (Ct) represents the transcript level of the mRNA. The stability of Ct values plays a crucial role in housekeeping gene selection: the level of expression of a gene depends on its Ct value; the lower the Ct value, the higher the expression level, and vice versa. RT-qPCR was employed in the evaluation of the expression patterns of eight internal control genes under diverse conditions. Figure 1 shows that the Ct values of the eight housekeeping genes ranged from 17.93 (*TUB*) to 29.7 (*ACTR*), with most between 22 and 27. According to further studies on the distribution of Ct values, the Ct values of the eight housekeeping genes were different under different conditions. Under different sex conditions, *GAPDH* expression levels were higher, and the Ct values of the eight housekeeping genes were mostly concentrated between 24 and 28, but the Ct values of *GAPDH* genes were concentrated around 22. Under different somites conditions, *GAPDH* expression levels were higher, and the Ct values of the eight internal reference genes were mostly concentrated between 22 and 26, while the Ct values of *GAPDH* genes were concentrated around 21.5. The expression levels of *TUB* were higher under different temperatures, diet, and pesticide conditions, but the Ct values of *TUB* genes were concentrated in 19, 21, and 21, respectively. Specifically, *RPL18* and *TUB* had mean Ct values in the sex of 27.06 and 23.23, respectively, but under temperature conditions, they had mean Ct values of 22.81 and 19.55, respectively. In the sex condition, the Ct mean value of the *ACTR* was 28.86, while it was 25.61 under situations involving various other conditions. Overall, *TUB* was the most abundant gene, and *ACTR* was the least expressed gene.

### 3.3. geNorm Analysis

**Figure 2:**
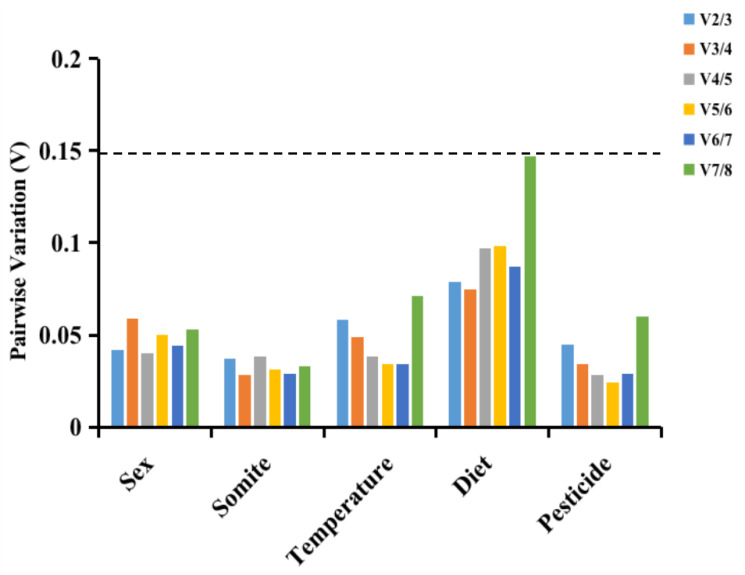
Evaluation of the optimum amount of housekeeping genes under different experimental conditions of *L. invasa.* When the V value is less than 0.15, there is no need to add additional internal reference genes for normalization.

**Figure 3:**
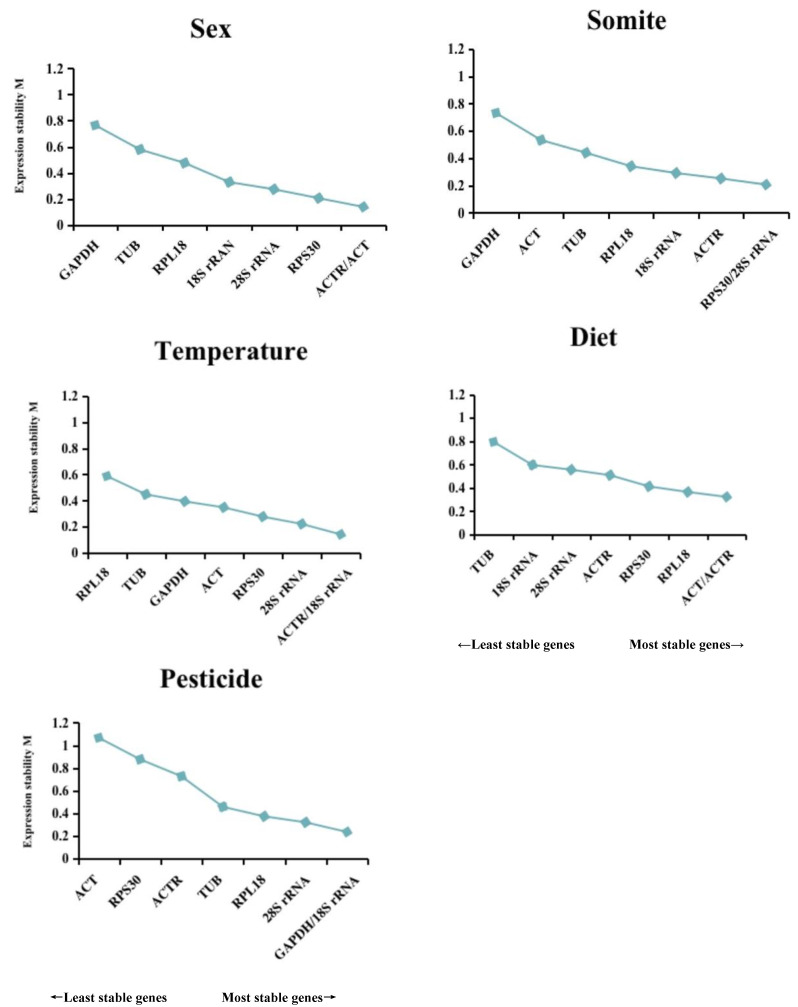
The expression stability values (M) of the eight housekeeping genes were verified by the geNorm program. The least stable genes with higher M values are on the left side, and the steadiest genes with lower M values are on the right.

geNorm evaluated the stability of each of the eight possible internal benchmark genes using the M value. The smaller the value of M, the more reliable the expression of the gene [10,19]. All eight housekeeping genes had M values lower than 0.15 in each setting, as shown in Figure 2, and they all varied in their levels of stability between settings. The fittest genes for different sexes and diets are *ACTR* and *ACT*, which have the same M value. The housekeeping gene, *18S rRNA*, demonstrated excellent stability under temperature and pesticide conditions. In sex and somite, *GAPDH* was the most unreliable housekeeping gene. geNorm software also gives data on the perfect amount of benchmark genes to be tested based on the pair-wise variance between ranking genes (V_n/n + 1_). Typically, V_n/n + 1_ is utilized to decide whether more housekeeping genes are required [10,19]. In V_n/n + 1_ > 0.15, case n + 1 housekeeping genes must be utilized. Conversely, just n housekeeping genes are necessary [10,19]. Figure 2 indicated, to properly normalize these treated samples, that only the two housekeeping genes were required, as evidenced by the V_2/3_ values for the sex (0.042), somite (0.037), temperature (0.058), diet (0.079), and pesticide (0.045) samples being less than 0.15. The use of two benchmark genes is preferred in gene quantification research. As shown in Figure 3, geNorm analysis was utilized to identify the fittest benchmark gene pairs under different settings. Results revealed that *ACT+ACTR* exhibited the best stability under various sex conditions, while *RPS30*+*28S rRNA* demonstrated superior performance in somite-related analyses. The two genes with the most excellent stability under diverse temperature settings were *ACTR*+*18S rRNA*. Meanwhile, *ACT+ACTR* was the most reliable housekeeping gene across different diet settings. Lastly, *GAPDH*+*18S rRNA* showed the most excellent stability under various pesticide conditions.

### 3.4. Comparative ∆Ct Analysis

**Figure 4:**
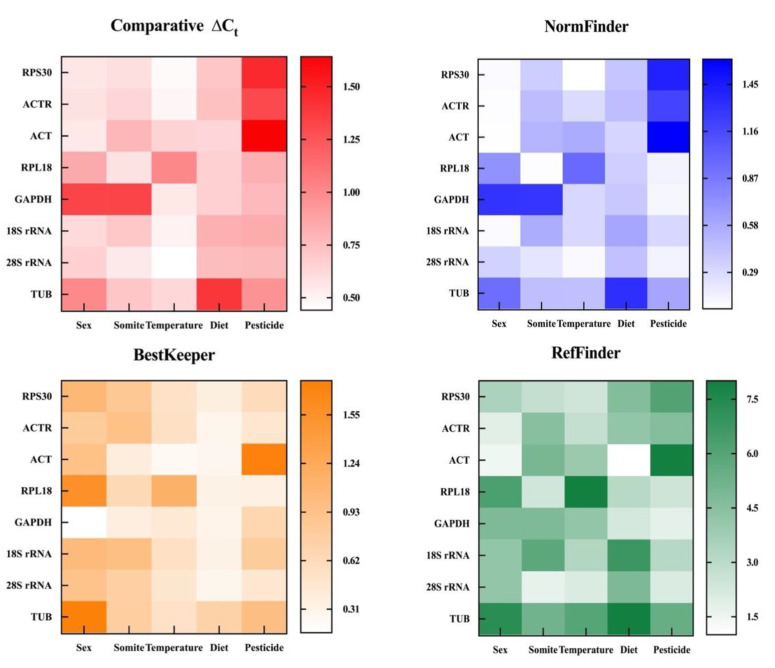
The eight housekeeping genes were used as the subject of stability assessments by NormFinder, BestKeeper, comparative ∆Ct, and RefFinder. The pane’s smaller value and lighter hues show the reference gene’s stability.

In this approach, gene expression stability is evaluated by calculating each gene’s mean and standard deviation (SD) value. Comparative ∆Ct analysis revealed that *GAPDH* had the most unstable expression across different sex and somite conditions. The *ACT* was the best housekeeping gene for gene normalization between sex and various dietary conditions. The most reliable housekeeping gene was *28S rRNA* for various somites, temperatures, and pesticide circumstances (Figure 4 and Table 2).

### 3.5. NormFinder Analysis

NormFinder software directly assesses the reliability of internal benchmark genes based on intra- and inter-group differences, with lower values indicating more excellent stability [20]. Figure 4 and Table 2 display the steadiness of the E values of benchmark genes under each treatment. Results showed that *RPL18* was the most trustworthy benchmark gene across different somites, while *ACT* exhibited superior performance in sex and diet-related analyses. Moreover, *RPS30* was optimal under varying temperature conditions. Lastly, *GAPDH* was identified as the most applicable under multiple pesticide environments.

### 3.6. BestKeeper Analysis

By measuring the standard deviation (SD), coefficient of variation (CV), Pearson correlation coefficient (CC), and *p* (probability value) of the Ct values, BestKeeper evaluated the steadfastness of gene expression. Less SD and CV indicate a better level of gene expression. The gene was deemed unacceptable for the benchmark genes when SD > 1 or *p* > 0.05 [21]. The analyses’ findings are presented in Table 3 and Figure 4. The top-ranked gene under different sex conditions was *GAPDH*, but its *p* value was higher than 0.05, disqualifying it from internally serving as a reference gene. *RPS30*, *RPL18*, *18S rRNA*, and *TUB* were four other genes whose SD values were higher than 1, disqualifying them from being used as benchmark genes. Finally, an evaluation of stable internal benchmark genes under various sexes was *ACTR* > *28S rRNA* > *ACT*. The eight genes under different somite’s SD and *p* values complied with the reference genes’ norms, and a stability ranking was *GAPDH* > *ACT* > *RPL18* > *28S rRNA* > *TUB* > *RPS30* > *ACTR* > *18S rRNA*. Even though *ACT* was the most precise internal benchmark gene, under different temperature states, its *p* value was higher than 0.05, which was unsuitable as a benchmark gene. Meanwhile, the SD value of *RPL18* was more than 1, which was also problematic as an internal benchmark gene. The remaining six’s stability order internal reference genes were *GAPDH* > *28S rRNA* > *RPS30* > *TUB* > *ACTR* > *18S rRNA*. Under various diet conditions, only the SD and *p* values of *RPS30* and *TUB* met the requirements. They were suitable for the internal reference genes, having a stability score of *RPS30* > *TUB*. The *p* values of *GAPDH*, *ACT*, *RPL18*, *28S rRNA*, *ACTR*, and *18S rRNA* were all greater than 0.05 and did not meet the requirements of the benchmark genes. The SD value of *ACT* was more than 1, which did not fulfil the standards of the reference genes under various pesticide circumstances. The remaining seven reference genes’ SD and *p* values were acceptable, and *RPL18* > *ACTR* > *28S rRNA* > *RPS30* > *GAPDH* > *18S rRNA* > *TUB*.

### 3.7. Comprehensive Ranking of Reference Genes

**Figure 5:**
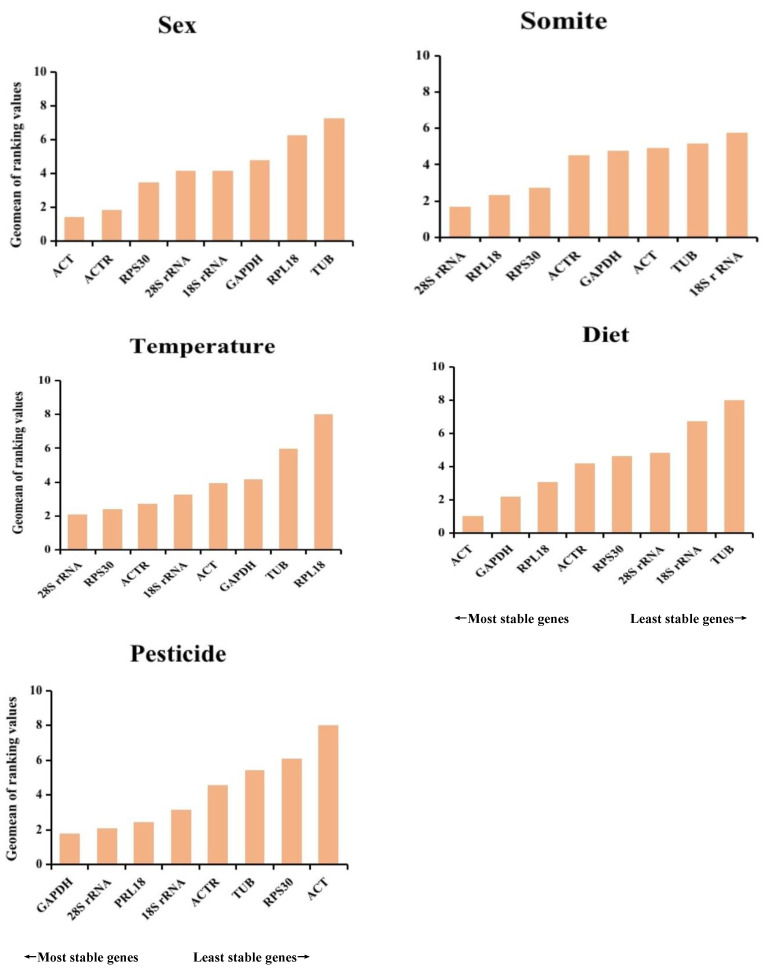
Eight housekeeping genes for *L. invasa* were ranked for stability under various treatment conditions using RefFinder.

Using the online tool RefFinder (https://blooge.cn/RefFinder/type=reference, accessed on 18 January 2023), the combined stability ranking of the benchmark genes was determined to lessen the effects of a single algorithm’s limitation. The geometric mean used to rank genes was calculated, and the stability increased as the geometric mean decreased [28]. Figure 5 displays that the two most trustworthy housekeeping genes for various sex conditions were *ACT* and *ACTR*; *28S rRNA* and *RPL18* were thought to be the best combination under different somites; *28S rRNA* and *RPS30* were the most appropriate housekeeping genes under various temperature circumstances; the two housekeeping genes with the highest levels of stability were *ACT* and *GAPDH* under diverse dietary circumstances; and *GAPDH* and *28S rRNA* were the most trustworthy housekeeping genes under diverse pesticides. Under most circumstances, *TUB* is the housekeeping gene that is the most unreliable.

### 3.8. Verification of Reference Genes

**Figure 6:**
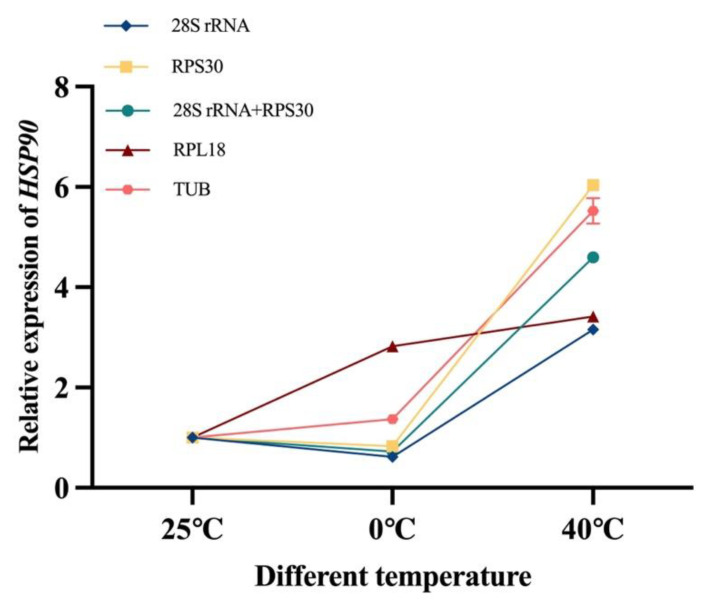
Relative expression levels of *HSP90* under diverse temperatures in adult *L. invasa* using different housekeeping genes.

The heat shock protein 90 of *L. invasa* was utilized as the objective gene to attest stability of benchmark genes. In this study, two of the most reliable housekeeping genes (*28S rRNA* and *RPS30*) and two of the least reliable reference genes (*RPL18* and *TUB*) were chosen to be evaluated under diverse temperature states (25 °C was used as a control). The gene expression trends consistently used *28S rRNA* and *RRS30* as housekeeping genes in Figure 6. Specifically, the expression of *HSP90* at 0 °C was lower than that at 25 °C, and at the same time, that at 40 °C was significantly higher than that at 25 °C and 0 °C. Overall, gene expression results were consistent when both *28S rRNA* and *RPS30* were used individually or combined as housekeeping genes. The expression of *HSP90* at 0 °C was greater than that at 25 °C when we used *RPL18* and *TUB* as housekeeping genes, in contradiction of the consequences obtained with *28S rRNA* or *RRS30* as housekeeping genes.

## 4. Discussion

In this study, the expression steadiness of eight benchmark genes was evaluated under different sexes, adult somites, temperature treatments, different dietary conditions, and pesticide treatments. According to this finding, no benchmark gene was suitable for each condition. Using a gene consistent across all experimental conditions as a control is much better than using a gene previously found to be highly consistent under only a very limited number of conditions, because there may be a nuisance variable that makes a gene that looks like a good fit in a given situation turn out to be a bad control. However, it is impossible to accurately predict any gene’s expression under a specific set of conditions. Therefore, to select an appropriate reference gene for an RT-qPCR experiment, the recommended strategy is to choose several candidate genes and evaluate their expression levels in various experimental conditions and treatments. This approach will identify those genes that exhibit the most stable expression levels under different test conditions and thus serve as the most appropriate control genes in the experiment. However, this topic has yet to get much attention, and its significance does not seem adequately understood. Because the option of reference genes varies drastically, insects of the same species, in terms of insect morphology, developmental stage, temperature, sex, and diet conditions, suggest that there is also no absolute generality between benchmark genes for the same species [29,30,31,32]_._ For instance, *PGK* and *RPL13* are acceptable internal housekeeping genes in *Cnaphalocrocis medinalis* under different sexes [31]. *PGK* and *EF1α* are stable housekeeping genes expressed in *Cnaphalocrocis medinalis* larvae under different temperature conditions [31]. Thus, for specific experimental treatments of *L. invasa*, it is indispensable to select appropriate housekeeping genes.

So far, many Hymenoptera species have found trustworthy housekeeping genes under different conditions, including *Solenopsis invicta* [32], *Aphidius gifuensis* [33], and *Apis mellifera* [34]. Nevertheless, reference genes for *L. invasa* have not been chosen or verified in earlier studies. *L. invasa* is an important pest of the genus *Eucalyptus*, mainly affecting seedlings and young forests. It forms galls on leaf veins, petioles, and current year branches, which in severe cases can lead to seedling mortality, up to 100% plant damage in young stands, and a significant reduction in yield in affected stands. Given future dispersal trends and changing environments, we should investigate molecular pathways for better management and control measures. The stability of eight regularly used internal housekeeping genes is explored in this study using various algorithms under five different experimental circumstances.

The commonly used statistical-analysis-based algorithms for evaluating the suitability of internal benchmark genes include geNorm, NormFinder, BestKeeper, and comparative ΔCt. The rankings generated in this research by geNorm, NormFinder, and comparative ΔCt are more like one another and different from the orders obtained by BestKeeper. For instance, *ACT* was the fittest in different sexes according to the results of geNorm, NormFinder, and comparing ΔCt. However, according to BestKeeper analysis, *ACT* was the fourth trustworthy housekeeping gene in different sexes. In contrast, *GAPDH* took the top spot in the BestKeeper analysis. In *Nitraria tangutorum* [35], relative differences between BestKeeper and other programs have also been noted. These differences probably result from these systems’ different algorithms [35]. RefFinder is a comprehensive evaluation tool that generates stability scores by estimating the geometric mean of internal control genes to lessen the effects of a single algorithm’s limitations [36]. Many species, including *Neoceratitis asiatica* [18] and *Anastatus japonicus* [17], were studied using a similar strategy. The expression of *HSP90* under different temperature treatments was evaluated to authenticate our findings. After normalization with *28S rRNA*+*RPS30* and *RPL18*+*TUB*, the expression results of *HSP90* were different. This outcome indicates that it is essential to pick the appropriate benchmark gene to normalize the expression of the target gene. Some studies reported that accurate RT-qPCR results required two or more stable internal benchmark genes [37,38]. The geNorm algorithm can compute the perfect amount of internal control genes for standardization based on whether V_n/n+1_ is less than 0.15 [10,19]. Only two housekeeping genes were used as a benchmark for qRT-PCR in this research to increase the accuracy of the data.

Actin genes can encode the cytoskeleton and regulate the structural integrity of cells [38]. Actin genes include *ACT8* and *ACT11*, which we have long used as housekeeping genes [39]. In fact, under various diapause states and in different tissues, *Anastatus japonicus* [17] displayed that *ACTIN* was the most reliable, consistent with earlier findings on *Locusta migratoria* [40] and *Spodoptera litura* [41]. In this study, *ACT* expresses steadily in most cases. Moreover, five algorithms assessed *ACT* as the perfect housekeeping gene under various diet circumstances. The validity of *ACT*, which was previously used in the study as a housekeeping gene for Hymenoptera, was further validated by this work. Still, our study found that the recommended amount of benchmark genes is two under diverse conditions. However, some studies on *L. invasa* used a single benchmark gene in a previous study. Therefore, we propose using two benchmark genes for normalization in future molecular experiments on *L. invasa*. Under varied sex and diet conditions, the most trustworthy housekeeping gene for *L. invasa* was *ACT*, but it was less reliable under different pesticides and somites. In the same species, a housekeeping gene may react differently to various conditions, like Kentucky bluegrass [42] and *Klebsormidium nitens* [43]. In conclusion, depending on the species, tissue, and treatment, it is frequently required to choose specific housekeeping genes.

## 5. Conclusions

As a result, the steadiness of the eight benchmark genes was tested using five trustworthy approaches in various experimental conditions. Regardless of which algorithm was used to assess the reference genes, *ACT* was most stable under different dietary conditions. After ranking the housekeeping genes’ stability, geNorm was used to calculate V_n/(n + 1)._ Two housekeeping genes were required as a benchmark for RT-qPCR to improve the trustworthiness of the qRT-PCR results. Five algorithms were combined to screen for the best combination of housekeeping genes under different conditions. These combinations included *ACT* and *ACTR* for different sexes, *28S rRNA* and *RPL18* for different somites, *28S rRNA* and *RPS30* for various temperature treatments, *ACT* and *GAPDH* for various diet treatments, *GAPDH* for various pesticide conditions, and *28S rRNA*. This finding will improve the precision of target gene expression quantification and lay the foundation for the study of gene function and the molecular mechanisms involved in *L. invasa* resistance. Despite efforts to identify stable internal control genes for use in gene expression studies, it is important to note that there is no one-size-fits-all solution. The housekeeping genes recommended in this study demonstrate high stability and accuracy under specific experimental conditions, but their applicability cannot be assumed across all settings.

## Figures and Tables

**Table 1:** Reference and target genes’ effectiveness, primer sequences, and product sizes.

Gene Name	Gene Symbol	Primer Sequences (5′ to 3′)	Tm (°C)	Length (bp)	Efficiency (%)	R^2^
Ribosomal protein S 30	*RPS30*	F:AACGCCAAAGGTTGAGAAGC	54	141	95.6	0.991
		R:TATGGGTTAGGGTTGGCGTT				
Actin-related protein	*ACTR*	F:GCAAAACACAGCCACCACT R: TGCCAAACCTAACAATCCGA	54	138	99.4	0.993
18S ribosomal RNA	*18S rRNA*	F:CCAGTGCAAAATGAAACGCC R:CATCGGGTGTGGATCAGGAT	55	165	99.7	1.000
Actin	*ACT*	F: CTACTGTACCACTCCGTCGC R:GGTCATTGGAAGTGGAGGCA	55	300	102.1	0.996
Ribosomal protein L 18	*RPL18*	F:ATGAAGAAGCCAGGACGTA R:CTTGGATCAGCACGGTCTTG	55	214	97.6	0.995
Glyceraldehyde-3-phosphate dehydrogenase	*GAPDH*	F:GCGATCAAGGCTAAGGTCAA	55	169	99.2	0.990
		R:ACGAGATGAGCTTGACGAAC				
28S ribosomal RNA	*28S rRNA*	F: GCCTCCCATCTGAAGACCTT R:GGTCGTGTGGTATTGAAGGC	55	179	101.2	1.000
B-tubulin	*TUB*	F:TACTGGATTCAAGGTCGGCA R: ACCTTCCTCCATACCTTCGC	56	205	98.8	0.996
Heat shock protein 90	*HSP90*	F: AGCTCTCTGAACTTCTGCGT R: GAAACCACGCTTCCTCACTC	57	176	99.1	0.997

**Table 2:** Ranking of the *L. invasa* housekeeping genes under various circumstances.

Condition	Rank	∆Ct		geNorm		NormFinder		BestKeeper	
Sex	1	*ACT*	0.542	*ACT/ACTR*	0.139	*ACT*	0.069	*GAPDH*	0.160
	2	*RPS30*	0.556	*-*	-	*ACTR*	0.075	*ACTR*	0.815
	3	*ACTR*	0.576	*RPS30*	0.206	*18S rRNA*	0.087	*28S rRNA*	0.918
	4	*18S rRNA*	0.614	*28S rRNA*	0.274	*RPS30*	0.093	*ACT*	0.933
	5	*28S rRNA*	0.662	*18S rRNA*	0.329	*28S rRNA*	0.334	*18S rRNA*	1.017
	6	*RPL18*	0.849	*RPL18*	0.476	*RPL18*	0.718	*RPS30*	1.055
	7	*TUB*	0.999	*TUB*	0.579	*TUB*	0.941	*RPL18*	1.557
	8	*GAPDH*	1.325	*GAPDH*	0.765	*GAPDH*	1.293	*TUB*	1.767
Somite	1	*28S rRNA*	0.540	*RPS30/28S rRNA*	0.206	*RPL18*	0.072	*GAPDH*	0.366
	2	*RPL18*	0.575	*-*	-	*28S rRNA*	0.217	*ACT*	0.385
	3	*RPS30*	0.590	*ACTR*	0.251	*RPS30*	0.364	*RPL18*	0.639
	4	*ACTR*	0.633	*18S rRNA*	0.291	*TUB*	0.450	*28S rRNA*	0.757
	5	*18S rRNA*	0.699	*RPL18*	0.341	*ACTR*	0.468	*TUB*	0.773
	6	*TUB*	0.710	*TUB*	0.440	*ACT*	0.514	*RPS30*	0.850
	7	*ACT*	0.778	*ACT*	0.533	*18S rRNA*	0.557	*ACTR*	0.924
	8	*GAPDH*	1.328	*GAPDH*	0.732	*GAPDH*	1.283	*18S rRNA*	0.968
	1	*28S rRNA*	0.440	*ACTR/18S rRNA*	0.141	*RPS30*	0.065	*ACT*	0.227
	2	*RPS30*	0.462	*-*	-	*28S rRNA*	0.097	*GAPDH*	0.432
Temperature	3	*ACTR*	0.483	*28S rRNA*	0.221	*ACTR*	0.277	*28S rRNA*	0.476
	4	*18S rRNA*	0.496	*RPS30*	0.277	*18S rRNA*	0.295	*RPS30*	0.519
	5	*GAPDH*	0.542	*ACT*	0.348	*GAPDH*	0.297	*TUB*	0.530
	6	*TUB*	0.623	*GAPDH*	0.394	*TUB*	0.440	*ACTR*	0.532
	7	*ACT*	0.648	*TUB*	0.448	*ACT*	0.564	*18S rRNA*	0.535
	8	*RPL18*	1.009	*RPL18*	0.588	*RPL18*	0.965	*RPL18*	1.139
Diet	1	*ACT*	0.631	*ACT/GAPDH*	0.322	*ACT*	0.317	*ACT*	0.267
	2	*GAPDH*	0.662	*-*	-	*RPL18*	0.356	*ACTR*	0.276
	3	*RPL18*	0.662	*RPL18*	0.364	*GAPDH*	0.395	*28S rRNA*	0.277
	4	*RPS30*	0.709	*RPS30*	0.412	*RPS30*	0.413	*GAPDH*	0.294
	5	*ACTR*	0.734	*ACTR*	0.508	*28S rRNA*	0.439	*RPL18*	0.318
	6	*28S rRNA*	0.755	*28S rRNA*	0.555	*ACTR*	0.459	*18S rRNA*	0.319
	7	*18S rRNA*	0.811	*18S rRNA*	0.596	*18S rRNA*	0.597	*RPS30*	0.373
	8	*TUB*	1.390	*TUB*	0.794	*TUB*	1.326	*TUB*	0.719
Pesticide	1	*28S rRNA*	0.765	*GAPDH/18S rRNA*	0.235	*GAPDH*	0.118	*RPL18*	0.339
	2	*GAPDH*	0.768	*-*	-	*28S rRNA*	0.135	*ACTR*	0.460
	3	*RPL18*	0.814	*28S rRNA*	0.320	*RPL18*	0.136	*28S rRNA*	0.460
	4	*18S rRNA*	0.838	*RPL18*	0.373	*18S rRNA*	0.306	*RPS30*	0.607
	5	*TUB*	0.962	*TUB*	0.457	*TUB*	0.603	*GAPDH*	0.673
	6	*ACTR*	1.301	*ACTR*	0.727	*ACTR*	1.189	*18S rRNA*	0.813
	7	*RPS30*	1.452	*RPS30*	0.877	*RPS30*	1.401	*TUB*	0.976
	8	*ACT*	1.641	*ACT*	1.068	*ACT*	1.604	*ACT*	1.752

**Table 3:** BestKeeper’s assessment of the steadiness of eight housekeeping genes.

		Gene							
Conditions		*RPS30*	*ACTR*	*ACT*	*RPL18*	*GAPDH*	*18S rRNA*	*28S rRNA*	*TUB*
Sex	SD (CP)	1.06	0.81	0.93	1.56	0.16	1.02	0.92	1.77
	CV (CP) %	4.19	2.82	3.78	5.75	0.73	3.76	3.41	7.61
	CC (r)	0.991	0.995	0.991	0.991	0.001	0.981	0.942	0.999
	P	0.001	0.001	0.001	0.001	0.904	0.001	0.005	0.001
Somite	SD (CP)	0.85	0.92	0.39	0.64	0.37	0.97	0.76	0.77
	CV (CP) %	3.83	3.61	1.68	2.50	1.69	3.99	3.05	3.48
	CC (r)	0.997	0.965	0.720	0.964	0.001	0.939	0.988	0.877
	P	0.001	0.001	0.029	0.001	0.001	0.001	0.001	0.002
Temperature	SD (CP)	0.52	0.53	0.23	1.14	0.43	0.54	0.48	0.53
	CV (CP) %	2.34	2.05	1.07	4.99	2.12	2.24	1.93	2.71
	CC (r)	0.959	0.878	0.525	0.964	0.876	0.87	0.951	0.857
	P	0.001	0.002	0.147	0.001	0.002	0.002	0.001	0.003
Diet	SD (CP)	0.37	0.28	0.27	0.32	0.29	0.32	0.28	0.72
	CV (CP) %	1.62	1.02	1.19	1.33	1.38	1.29	1.09	3.44
	CC (r)	0.685	0.001	0.232	0.442	0.097	0.174	0.178	0.659
	P	0.014	0.412	0.468	0.150	0.764	0.588	0.580	0.020
Pesticide	SD (CP)	0.61	0.46	1.75	0.34	0.67	0.81	0.46	0.98
	CV (CP) %	2.58	1.68	7.23	1.38	2.99	3.12	1.76	4.60
	CC (r)	0.001	0.001	0.989	0.922	0.986	0.965	0.988	0.979
	P	0.002	0.001	0.001	0.001	0.001	0.001	0.001	0.001

## Data Availability

The data presented in this study are available on request from the corresponding author.
